# 

**Published:** 1897-10-02

**Authors:** 


					THE hospital. ABSOLUTELY PURE, therefore BEST. II-Qct^2nd,_189L
CADBURY'S WW ?**? CADBURY^RY
OOOOA HP" W ST1 Ktfisew GENERALS OFFICE
'7^S?-lSrP?r',F*,P""?' ^ ^ NO |LKALIEg^ED^
fltf/CH HOSf LTAJ,
No. 575. Vol. XXIII. E3*5*?] Saturday,
Oct. 2nd, 1897.
[feubseription^Tcirms,] pE)ICE TWOPENCE.

				

